# How can we make better health decisions: a Best Buy for all? Commentary based on discussions at iDSI roundtable on 2 ^nd^ May 2019 London, UK

**DOI:** 10.12688/gatesopenres.13063.2

**Published:** 2020-01-21

**Authors:** Niki O'Brien, Ryan Li, Wanrudee Isaranuwatchai, Saudamini Vishwanath Dabak, Amanda Glassman, Anthony J. Culyer, Kalipso Chalkidou

**Affiliations:** 1 Global Health and Development Group, Imperial College London, London, UK; 2 Centre for Excellence in Economic Analysis Research (CLEAR), Li Ka Shing Knowledge Institute, St. Michael’s Hospital, Toronto, Canada; 3 Institute of Health Policy, Management and Evaluation, University of Toronto, Toronto, Canada; 4 Health Intervention and Technology Assessment Program (HITAP), Nonthaburi, Thailand; 5 Center For Global Development, Washington DC, USA; 6 Centre for Health Economics, University of York, York, UK; 7 Center For Global Development, London, UK

**Keywords:** health technology assessment, global health, healthcare, financing, healthcare financing, economic evaluation, international development, donor, donor transition, international decision support initiative, health services, universal health coverage, health system, health system strengthening, HTA institutionalization, evidence-informed priority setting, priority setting, resource allocation, purchasing

## Abstract

The World Health Organization (WHO) resolution calling on Member States to work towards achieving universal health coverage (UHC) requires them to prioritize health spending. Prioritizing is even more important as low- and middle-income countries transition from external aid. Countries will have difficult decisions to make on how best to integrate and finance previously donor-funded technologies and health services into their UHC packages in ways that are efficient and equitable, and operationally and financially sustainable.

The International Decision Support Initiative (iDSI) is a global network of health, policy and economic expertise which supports countries in making better decisions about how best to spend public money on healthcare. In May 2019, iDSI convened a roundtable entitled *Why strengthening health systems to make better decisions is a Best Buy*. The event brought together members of iDSI, development partners and other organizations working in the areas of evidence-informed priority-setting, resource allocation, and purchasing. The roundtable participants identified key challenges and activities that could be undertaken by the broader health technology assessment (HTA) community:

•           to develop a new publication package on premium estimation and budgeting, actuarial calculations and risk adjustment, provider payment modalities and monitoring of quality in service delivery

•           to call on the WHO to redouble its efforts in accordance with the 2014 Health Intervention and Technology Assessment (HITA) World Health Assembly resolution to support countries in developing priority setting and HTA institutionalization, and to lead by example through introducing robust HTA processes in its own workings

•             to develop a single Theory of Change (ToC) for evidence-informed priority setting, to be agreed by the major organizations working in the areas of priority setting and HTA.

## Introduction

The World Health Organization (WHO) resolution calling on Member States to work towards achieving universal health coverage (UHC) has increased the need for prioritizing health spending ^1^. Prioritizing is even more important as low- and middle-income countries (LMICs) transition from external aid. By 2022, twenty-four countries are projected to be in transition ^2^. Countries will have to make difficult decisions on how best to integrate and finance previously donor-funded technologies and health services into their UHC packages, how to finance them, and how to identify and make any necessary trade-offs between competing health priorities to ensure that high-quality, affordable access to healthcare is provided to the population in ways that are efficient and equitable, and operationally and financially sustainable.

But how might priority-setting best be done? Health technology assessment (HTA) is a multi-disciplinary exercise for assessing the clinical and cost-effectiveness of technologies in the form mainly of programs of health (and sometimes social) care, together with their associated structural, procedural and implementation arrangements ^3^. The WHO has argued that HTA should be a clear part of the priority-setting process and be an important means through which UHC can be achieved and secured. The WHO defines the scope of technologies which can be evaluated using HTA as “medicines, medical devices, vaccines, procedures and systems” ^4^.

The International Decision Support Initiative (iDSI) is a global network of health, policy and economic expertise which supports countries in making better decisions about how best to spend public money on healthcare ^5^. Funders of the network include the Bill & Melinda Gates Foundation (BMGF) and the UK Department for International Development (DFID). In May 2019, iDSI convened a roundtable ^1^ discussion in London, UK, entitled *Why strengthening health systems to make better decisions is a Best Buy*. The event brought together members of the iDSI network, its development partners, and other organizations working on evidence-informed priority-setting, resource allocation and purchasing. Discussions explored the priority-setting challenges that governments in Africa and Asia face and how HTA can be a means of strengthening health systems, why investment in evidence-informed priority-setting is a Best Buy for development partners, and how organizations working in this area might best work together. The roundtable participants identified several initiatives for the broader HTA community to extend country-led capacity-building in HTA and to foster deeper collaboration within the community.

## Why is HTA not yet routinely adopted as a tool for strengthening health systems for UHC?

### Confusion over definitions and terms

Prevailing unfamiliarity with the vocabulary of HTA by stakeholders was identified as a notable challenge to the institutionalization of HTA as a means of setting priorities. This is hardly surprising since the HTA community itself is still struggling with definitions. In 2019, a task group was set up to bring together several organizations working on HTA, including the WHO, to develop an agreed definition that codified the many existing definitions and reflected the “current and emerging realities of HTA” ^6^. The task group proposed the definition:

- A multidisciplinary process that uses explicit and scientifically robust methods to assess the value of using a health technology at different points in its lifecycle. The process is comparative, systematic, transparent and involves multiple stakeholders. The purpose is to inform health policy and decision-making to promote an efficient, sustainable, equitable and high-quality health system ^6^.

Roundtable participants elaborated that the definition must be clarified in order to reduce confusion concerning the *scope* of HTA, the *process* of priority setting, the *terms of engagement with stakeholders* and the *selection of topics* for evidence review. It was emphasized that HTA is more than a merely technocratic exercise. At its best it is a bridge joining professional technical assessment and political policy decision-making as a fully integrated whole. Assessment tools should therefore be designed to make clear the nature of the decisions that need to be taken, and the policy decision-making processes that would ensure that critically important political elements could be taken into account This is more than a matter of defining HTA, for it must include systematic consideration of context, scope and process, all of which will be conditioned by local history, culture and politics, but which might also need modification and adaptation if the objectives of HTA and UHC are to be delivered. HTA, then, was seen as both the portfolio of analytical tools for assessment (the “assessment” tools) and the broader range of topics that define context. This includes objectives, barriers, conflicting interests that, taken together, amount to a kind of “political economy” of healthcare prioritization. On this view, while the technical attributes of “assessment”, like the meanings of opportunity cost, evidence, effectiveness or model building, are common to all practical applications of HTA, in any specific application, the objectives, the weights attached to them, the scope of costs and outcomes and the historical and cultural context which may make decisions and their implementation more or less possible, are particular to that application. In this sense, “one size does not fit all”, there is no universally correct “perspective” of a study, and “cost-effectiveness” is a function of local circumstances: what is cost-effective in one place may or may not be cost-effective in another. Of course, there are differences in the methods applied across different contexts, and as such, there is value in having a reference case for HTA ^7^. Notably, the roundtable largely focused discussions on definitions and processes rather than methods. Roundtable participants concluded that, once consensus is reached in defining HTA, greater efforts should be devoted to communicating it authoritatively to stakeholders while concurrently striving to reach a consensus on the “political economy”: the elements that make HTA so much more than merely CEA, and the best ways of integrating those elements into the design of decision-making processes and the conduct of what is considered within them.

### The politics of HTA

Many countries have struggled with the rationing of health services to serve population needs ^8^. Evidence-informed priority-setting by HTA forces decision-makers and their advisors to be explicit when considering trade-offs when objectives clash, controversial value judgements emerge, and the means of implementation are limited. In so doing, HTA also provides credible justifications when decision-makers are called to account. It is important to note the judicialization of the right to health has played a role in challenging rational priority setting mechanisms, yet such challenges ensure that governments abide by international human rights ^9^. Dittrich *et al*. (2016) note that rational priority setting, based on evidence and with a focus on equity, is the best way to safeguard an ethical allocation of scarce healthcare resources ^9^. Without such processes decision-makers may find it easier to say “yes” when honesty requires a “no”. Such a decision, may avoid short term embarrassment, but is likely also to be damaging to health and, most probably, its distribution – and with little defensive armor remaining by way of dutiful adherence to principle and protocol, or to the evidence when the consequential unsustainable outcomes reveal themselves. However, with proper HTA processes, decisions that may be to some group’s disadvantage can be readily shown nonetheless to have considered all interests and made fair judgments, to have evaluated the evidence properly, consulted the country’s best experts, and followed procedure.

It is therefore important to make decision-makers aware of the purpose and power of HTA. Awareness of what HTA *is not* is no less important. HTA’s purpose is not to cut costs nor to cut services: only those that are a demonstrable waste. In fact, HTA helps drive efficient resource allocation, and at least in the case of the National Institute for Health and Care Excellence (NICE) in England and Wales, has also probably been critical in supporting (confidential) price discounts on a range of potentially very expensive technologies ^10, 11^. As such, the emphasis is on enhancing system efficiency and quality through HTA and HTA-related (e.g. Standard Treatment Guidelines (STGs)) evidence. Participants of the roundtable emphasized the importance of ensuring that everyone understands that HTA is there to make it easier for decision-makers to reject service improvements and innovation where needed. For example, HTA in India has been focused on improving the quality of healthcare services. Since 2009 iDSI has worked at the national and state level in India introduce HTA, develop institutional structures with clear goals, including the development of STGs ^12^.

Moreover, decision-makers should learn as a practical matter, that HTA, especially when combined with budget impact analysis, has been successfully used as a tool to inform price negotiations and efficient procurement ^13^. When apparently ‘cost-effective’ interventions appear unaffordable in a particular context, recalibrating the threshold or staging the roll out of the intervention based on budget impact concerns might be warranted. One example of this kind of analysis in action was a cost utility analysis of pneumococcal conjugate vaccines for the Philippine context ^14^. In addition to presenting incremental cost-effectiveness ratios (cost per QALY gained) that showed that PCV could be cost-effective, the results showed that universal vaccination would cost 1.6 to 1.8 times the budget of the existing national vaccination program. The analysis highlighted the interplay between price, cost-effectiveness and budget impact, and in particular the challenges presented by fragmented health system financing. Such analytical approaches can underpin governments’ and development partners’ “Best Buys” agendas with solid data and consistent arguments that make sense for a given context.

### Lack of understanding about how policymakers and payers can use HTA

**Figure 1.**
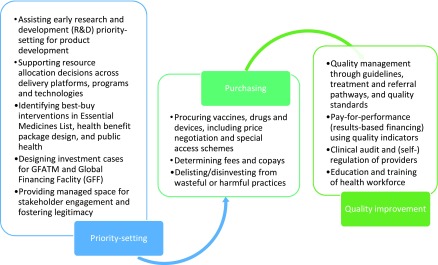
How health technology assessment (HTA) can be used to inform resource allocation and purchasing of health services interventions.

The need for enlarged research and analytical capacity, together with strengthened systems, is widespread in LMICs ^15^. The proliferation of global initiatives and organizations providing technical assistance is often uncoordinated, and managing aid programs can overwhelm governments and healthcare payers whose capacities are typically already overstretched ^16^. Further disorganization arises from there being multiple health packages, parallel decision-making, single topic donor funds, and client specific insurance schemes. A clearer narrative for systematic and open priority setting is needed. Participants discussed the need for developing a new publication which might build on *What’s In, What’s Out: Designing Benefits for Universal Health Coverage* ^17^, that would address current issues facing the UHC movement, help link priority setting to upstream service financing, actuarial initiatives, and provide a means of engaging with governments and budget holders in practical activities such as procurement and regional purchasing budget settings. We offer examples of practical applications of HTA in Figure 1.

### Country-owned applications of global and local evidence

Another major challenge is getting traditional academic researchers and organizations, whose HTA role lies primarily in providing technical assistance, to work together to serve LMICs governments in a responsive, demand-driven manner. Their roles are of critical importance in the fields of research reviews, foreign evidence interpretation, model estimation and re-estimation, primary research, and training. Some of this support might usefully be organized regionally. International development partners are already investing in data collection and synthesis, notably through initiatives like the Disease Control Priories Network (DCPN), funded by BMGF, which promotes and assists in the use of economic evaluation for priority setting at global and country levels. DCPN most recently published *Disease Control Priorities, Third Edition*, a review of evidence on cost-effective interventions for addressing the burden of disease in LMICs ^18^. However, the next phase of work for DCPN and other organizations in the HTA community, should bring together academics, data and technical assistance to ensure that their work is locally owned, focused on the real needs of local policy, and that it exploits economies of scale and scope ^15^.

### Working towards sustainability and cohesion

The roundtable suggested that the WHO might become an explicit “knowledge broker”, connecting decision-makers with common demands for evidence or practical support and assistance when making difficult decisions. There are several excellent examples of data-driven collaboration including the CEVR Global Health Cost Effectiveness Analysis (GH CEA) registry, a database of CEA studies evaluating health interventions from around the world ^19^. Most recently, the WHO hosted Decide Health Decision hub, a platform established in June 2019 with the objective of hosting a virtual space for collaboration in data-driven health decision-making ^20^. The hub will be a useful platform for HTA, other economic and social evaluation, creating investment and disinvestment cases, or indeed any other policy territory that needs fair and transparent decision-making. The roundtable endorsed the development of the platform and called for all partners to support the WHO in its coordination efforts. The WHO could be a source of strong leadership and sustained support.

There is a secondary need to ensure that monitoring and evaluation mechanisms are in place for measuring and learning from mistakes. The changing configuration of global health financing means increasing reliance on loans and less on donor funding. Governments consequently need to take responsibility for the development of strong and sustainable health systems. They need to recognize and seek out individuals and organizations with the skills to assist where gaps exist. The roundtable endorsed the idea of a unified Theory of Change (ToC) for the major initiatives and organizations using HTA in priority setting. Developing a ToC involves working back from a long-term goal, like maximizing the impact of healthcare on health, mapping out the causal pathways (including outputs and intermediate outcomes) toward that goal, and the assumptions—factors that would facilitate or impede the achievement of the goal – that such modelling involves ^21^. The ToC would outline a mutually agreed goal related to HTA for UHC, with clear and measurable milestones and/or indicators to enable the monitoring of progression, with adjustments as one goes along. It could build on the existing iDSI Theory of Change, outlining how facilitation of effective partnerships, with purpose-driven institutional and technical capacity-building, results in better decision-making and sustainable health impact ^22^.

## Next steps and conclusions

Next steps identified by the roundtable participants were these:

- to develop a new publication package building on *What’s In, What’s Out*, linking upstream questions of premium estimation and budgeting, actuarial calculations and risk adjustment to downstream provider payment modalities, the monitoring of quality in service delivery, and the appropriate use of technology
- to call, in writing, for WHO to redouble its efforts following the 2014 Health Intervention and Technology Assessment (HITA) World Health Assembly resolution to support countries in developing priority setting and HTA institutionalization; to call also for leadership by example through introducing robust HTA processes in its own work: the development of Standard Treatment Guidelines (STGs), disease specific targets, and the Essential Medicines List (EML) as well as policy guidance documents
- to develop a convergent Theory of Change (ToC) for evidence informed priority setting, agreed by the major organizations working in the areas of priority setting and HTA to enhance coordination, alignment and minimize bureaucracy. This work could be facilitated through the WHO hosted Decide Health Decision hub, bringing together major HTA-focused organizations

## Data availability

No data are associated with this study.

## Notes

^1^ *Members of the iDSI roundtable included: Anthony J. Culyer (Chair), University of York, York, UK; Peter Baker, Global Health and Development Group (GHD), Imperial College London, London, UK; Maria Vittoria Bufali, University of Strathclyde, Glasgow, UK; Francoise Cluzeau, Global Health and Development Group (GHD), Imperial College London, London, UK; Kalipso Chalkidou, Center for Global Development (CGD); Global Health and Development Group (GHD), Imperial College London, London, UK; Saudamini Dabak, Health Intervention and Technology Assessment Program (HITAP) Thailand (Asia HTA Consortium), Nonthaburi, Thailand; Austen Davis, Norwegian Agency for Development Cooperation (Norad), Oslo, Norway; Samantha Diamond, Clinton Health Access Initiative (CHAI), Boston, USA; Suyai Ehlers, Global Health and Development Group (GHD), Imperial College London, London, UK; Sarah Garner, World Health Organization (WHO), Geneva, Switzerland; Amanda Glassman MSc, Center For Global Development (CGD); Javier Guzman, Management Sciences for Health, Medford, Massachusetts, USA; Martha Gyansa-Lutterodt, Ministry of Health Ghana, Accra, Ghana; Raph Hurley, Clinton Health Access Initiative (CHAI), Pretoria, South Africa; Wanrudee Isaranuwatchai, Health Intervention and Technology Assessment Program (HITAP) Thailand (Asia HTA Consortium), Nonthaburi, Thailand; Kjell-Arne Johansson, University of Bergen, Bergen, Norway; Jo Keatinge, Department for International Development (DFID), East Kilbride, UK; Ryan Li, Global Health and Development Group (GHD), Imperial College London, London; Rob Lloyd, Itad, Brighton, UK; Ingrid Miljeteig, University of Bergen, Bergen, Norway; Robyn Millar, University of Strathclyde, Glasgow, UK; Niki O’Brien, Global Health and Development Group (GHD), Imperial College London, London, UK; Trygve Ottersen, Norwegian Institute of Public Health (NIPH), Oslo, Norway; Francis Ruiz, Global Health and Development Group (GHD), Imperial College London, London, UK;, UK; Ingvil Saeterdal, Norwegian Institute of Public Health (NIPH), Oslo, Norway; Anna Vassall, London School of Hygiene and Tropical Medicine, London, UK; Damian Walker, Bill & Melinda Gates Foundation (BMGF), Seattle, USA; David Wilson, Bill & Melinda Gates Foundation (BMGF), Seattle, USA; Kun Zhao, China National Health Development Research Center (CNHDRC), Beijing, China.*

*The analysis of roundtable comments and subsequent discussion in this paper express the views of its authors. Any omissions are oversights from the authors rather the participants of the iDSI roundtable.*
