# Supplementary material for: How can we make better health decisions: a Best Buy for all? Commentary based on discussions at iDSI roundtable on 2 nd May 2019 London, UK
Source: Gates Open Res. 2020 Jan 21;3:1543. Originally published 2019 Sep 20. [Version 2] doi: 10.12688/gatesopenres.13063.2 (PMC6784300; doi:10.12688/gatesopenres.13063.2)
Supplement: Peer review history

# Peer review: Reviewer response for version 2

**DOI:** 10.21956/gatesopenres.14269.r28491

**Published:** 2020-02-20

**Authors:** Markus Haacker

**Affiliations:** 1 Department of Global Health and Population, Harvard T.H. Chan School of Public Health, Harvard University, Boston, MA, USA

Shortcomings to the previous version have been addressed, the article now consistently meets the required standards.

I confirm that I have read this submission and believe that I have an appropriate level of expertise to confirm that it is of an acceptable scientific standard.

# Peer review: Reviewer response for version 2

**DOI:** 10.21956/gatesopenres.14269.r28492

**Published:** 2020-01-22

**Authors:** Daniel A. Ollendorf

**Affiliations:** 1 Center for the Evaluation of Value and Risk in Health (CEVR), Tufts Medical Center, Boston, MA, USA

No other comments, approved as revised.

I confirm that I have read this submission and believe that I have an appropriate level of expertise to confirm that it is of an acceptable scientific standard.

# Peer review: Reviewer response for version 1

**DOI:** 10.21956/gatesopenres.14194.r27904

**Published:** 2019-10-10

**Authors:** Markus Haacker

**Affiliations:** 1 Department of Global Health and Population, Harvard T.H. Chan School of Public Health, Harvard University, Boston, MA, USA

The article summarizes discussions at a roundtable "Why strengthening health systems to make better decisions is a Best Buy." I did not participate the event and cannot comment on adequacy as a protocol of the discussions, my observations are based on the article alone.

 The article builds up to three recommendations or "next steps" - a publication package to develop and document practice of health technology assessment (HTA), a "call for WHO to redouble its efforts […]  to support countries in developing priority setting," and development of a "single Theory of Change." These recommendations follow clearly from the discussion.

 There are, however, a number of shortcomings which compromise the effectiveness of the letter:

1. The abstract is inadequate, and reads at first sight like an introduction. (Indeed, it turns out that is has been pasted and rearranged from the introduction.) This should be rewritten to communicate gist of discussions and recommendations.
2. The perspective - i.e., the dividing line between general remarks or exposition of a topic on one hand, and documentation of roundtable discussion on the other - is not always clear.
3. In the section on "confusion over definitions and terms" (paragraph starting " Participants of the roundtable noted ..." until end of section) I am puzzled by the contrast between the "professional technical assessment and professional policy decision-making." This, and the following paragraph in which this theme is developed, lacks a clear health systems perspective which would usefully complement and connect the themes developed.
4. The section on "Theory of Change" appears out of context, because the article here conflates challenges in application of HTA and the perspective of institutions driving the HTA agenda and embedding it in a broader health policy agenda. I propose to separate these perspectives and objectives more clearly.

I confirm that I have read this submission and believe that I have an appropriate level of expertise to confirm that it is of an acceptable scientific standard, however I have significant reservations, as outlined above.

# Peer review: Reviewer response for version 1

**DOI:** 10.21956/gatesopenres.14194.r27903

**Published:** 2019-10-08

**Authors:** Daniel A. Ollendorf

**Affiliations:** 1 Center for the Evaluation of Value and Risk in Health (CEVR), Tufts Medical Center, Boston, MA, USA

This Open Letter is a clear and concise statement that places good context around the use of health technology assessment (HTA) to support prioritization and financing of resources. Given that this was a summary of a round table session, perhaps not all of the following suggestions can be taken up, but they are intended to help clarify the discussion somewhat:

1. HTA definition: it is unclear whether the term "lifecycle" refers to the product or the HTA process. If the product, my belief is that there is really only one point in the lifecycle in which the value is explicitly considered. Other points (e.g., early scientific advice) are focused on evidence generation.
2. Politics of HTA: The discussion of how HTA should not be considered merely a vehicle for saying "no" is an opportunity to describe examples of costly interventions that are shown to be cost-effective given outsized benefits, cost-offsets, etc. This would more clearly show the benefit of value assessment as part of the HTA process.
3. Politics of HTA: Another example of use of HTA would be development of informative clinical guidelines (alluded to later).
4. Politics of HTA: This may not have been part of the discussions, but HTA's independence (arm's length relationship with government, multi-stakeholder approach) is one critical way to mitigate undue political influence.
5. Politics of HTA: "decision maker" should be "decision makers".
6. Policymaker/payer use of HTA: In the preceding paragraph, NICE's effects were described as increasing spending and putting "necessary pressure" on health budgets.  This may be true in some instances in LMICs as well, and may not necessarily always result in "realisable savings". Additional spending may be justified for health gain alone.
7. Working towards sustainability and cohesion: The goal of data-driven collaboration is a laudable one; are there examples outside of Decide that could be cited here?
8. Next steps and conclusions: In general, it is a good idea to spell out any acronyms that may not be clear to all readers, e.g. "HITA", "WHA", "EML", "STGs".
9. Next steps and conclusions: In the first bullet, it seems that "premier" should be "premium"?

I confirm that I have read this submission and believe that I have an appropriate level of expertise to confirm that it is of an acceptable scientific standard.

# Peer review: Reviewer response for version 1

**DOI:** 10.21956/gatesopenres.14194.r27902

**Published:** 2019-09-24

**Authors:** Hector E. Castro

**Affiliations:** 1 Pharmaceutical Economics & Financing, Management Sciences for Health, Arlington, VA, USA

The Open Letter is clear and relevant for the overall priority setting and health technology assessment community. A few comments below to potentially make the narrative more balanced:

- The confusion of terms is very relevant, perhaps could expand a little bit more on the confusion of methods? Not all countries apply the same principles for HTA, not all of them take the full CUA/CEA approach.
- The politics of HTA is of paramount importance for its successful implementation, however the paragraph seems a little bit short on the need to raise awareness among policymakers and get their buy in on more systematic priority setting processes. Also in many settings the historic lack of transparency of decision-making embedded in the context of health is being considered a human right has led to judicial intervention on behalf of the population posing an additional challenge to rational priority setting mechanisms.
- The section “Lack of understanding about how policymakers and payers can use HTA" mostly elaborates on the need for international coordination, but seems limited on the need to address the local capacity gaps of HTA "users" (i.e. policymakers, payers, etc.) so they can understand the potential use and limitations of HTA.
- Next steps and conclusions should include some notes on the need for capacity building of HTA users.

I confirm that I have read this submission and believe that I have an appropriate level of expertise to confirm that it is of an acceptable scientific standard.
